# A Case of Hysteria in a Young Lady

**Published:** 1887-03

**Authors:** W. M. Powell

**Affiliations:** Albany, Texas


### A CASE OF HYSTERIA IN A YOUNG LADY.

*By W. M. Powell, M. D., Albany, Texas.*

For Daniel's Texas Medical Journal.

HAVING read a report of a similar case in the American Journal of Obstetrics, it may be of interest to some of your readers to cite a case that came under my observation last summer.

Miss M. D., a beautiful blonde of seventeen summers, perfectly healthy, of good family history, presented herself at my office on the 18th day of July, complaining of feeling badly from constipation. I gave her a prescription for the trouble, which is very common in this country, and she left, stating that she would call again if she did not get better. The following afternoon her father sent for me to come quickly to see her, as she was "very bad." I found her suffering, apparently, from an intense pain in the left hypochondriac region, and on attempting to examine the affected side she would scream vociferously, and would not allow me to lay even the weight of my hand on her. The thermometer registered a normal temperature, her tongue clean, respirations normal, and her stomach and liver, as nearly as could be ascertained in an active, healthy condition—her bowels well opened from the laxative I had prescribed the day previous, but the paroxysmal pain seemed to be unbearable. An old lady friend of hers living near by was present, and had been with her during the afternoon, and I turned to her and asked concerning her kidneys and bladder. She informed me that she had voided large quantities of urine having the appearance of clear water. I then inquired very closely into the condition of her catamenia, and learned that everything in that particular was perfectly regular. I diagnosed hysteria, and communicated the fact to the old lady, who was intelligent enough to understand and appreciate the situation, and rid herself of the anxiety she had had of the young lady's condition. I prescribed an opiate, hoping to quiet her sufficiently, at least, to make a more thorough examination, thinking that there might possibly be some tenderness about the spleen, but it seemed to have no effect—she would cry out at regular intervals and grasp her side vigorously, but could not bear for me to touch her. I then attempted to produce complete anaesthesia with chloroform but, strange to say, failed. By this time her father and brothers (mother being dead) as well as numerous bystanders, became greatly alarmed, but I told them to be quiet, as there was no danger of her dying. Some three hours had elapsed after my arrival, when opisthotonos was well marked, pulse and respiration remaining normal, but a slight elevation of temperature accompanied with great thirst. She would at times cry out, with her face all contorted, that her great toe on the right foot was coming off, and insisted that some one should rub it, which, of course, was done. As a rule, the toe would be drawn downward and inward, but some times she would only imagine it so.

This state of things kept up until 10 o'clock P. M., when I sent for my friend, Dr. Moody, who came, and very promptly undertook to examine her, but was quite as unsuccessful as myself. We had no trouble in agreeing as to the nature of the case, but he thought there would be no difficulty in putting her under the influence of chloroform, and suggested that we try it again, to which I cheerfully consented. I had improvised a very good inhaler which I again charged and passed to him to apply, while I sat by to watch her pulse. At least one-third of a pound of chloroform had been consumed when the doctor thought she was thoroughly under its influence, and picked up her hand, letting it drop apparently lifeless by her side. He then proceeded to make the examination I had so signally failed to do, when, to his astonishment she grasped her side as before, opened her eyes, and screamed out "let me alone," as though the chloroform was nothing more than the oxygen she breathed every day. This interesting, but somewhat ludicrous state of affairs was suddenly changed by the appearance of a young gentleman friend of mine, who had been paying his respects to her for some time—his presence seemed to have a good effect for a while. Dr. M. and myself then repaired to an adjoining room for further consultation, and decided to give a large dose of chloral and bromide, combined. The doctor left, and I remained for a short time, giving only a part of the dose referred to, as she refused to swallow it all. She had been having choking spells, or rather spasm of the œsophagus which seemed to be growing more frequent by this time since the young man would quickly raise her to a semi-recumbent position, rub her face, etc. I finally told him if he would not molest her she would do better. I then told her that she could go to sleep if she would only try, and bade her good-night, telling her father as I passed out of the door, that unless she got a great deal worse than she ever had been, not to send for me before morning; that she imagined she was in a much worse condition than she really was. So I was not disturbed any more during the night. I called again at 6 o'clock next morning and learned that she had passed the remainder of the night quietly, but with the approach of day and the stir and bustle incident thereto, the attacks grew quite as bad as they were in the evening.

I withdrew everything in the shape of medicine, ordered her a plate of soup, which she relished. I then darkened the room and positively forbade anyone entering it during the day but her father and one lady friend. Of course Mr. —— was admitted after business hours, for he proved to be the best nurse of any. This was the course I pursued for two days and nights, and she made a good recovery. In November I treated the same party through an attack of the so-called "slow fever" peculiar to this country, without there being the slightest manifestation of hysterics. There was, at times, more or less tympanites, too, all over abdominal region, and on percussing the parts, she quietly submitted without a word or recoil.

Now I consider that the case presented two interesting features: First, her age, social standing, and extremely healthy condition; second, the disposition to repel anaesthetics, narcotics, &c. We know that there are persons more easily anæsthetised than others; that we frequently meet with cases where the different forms of opium do not produce the desired effect. But when we, after having persisted in the use of such drugs, fail to accomplish anything at all, we very naturally want to know the reason why, and give such a case publicity.

Never having met with this peculiar affection in the young unmarried woman before, I will not venture an opinion as to the probable cause of it.

I believe the manifestations of hysteria are attributable, as a rule, to a functional derangement of the brain and spinal cord; and at the same time I believe that there may be other phenomena to excite it, as in this case. There was nothing before the attack, or has there been anything since, that would lead one to infer that there was anything wrong with her mental faculties. It is true that the mental exaltation during the paroxysm denoted irritability of some character; but where it come from, or what produced it I am not able to say. Now if there had been some functional disturbance of the uterus or its appendages, or any other organ, it would be different; but as I said in the outset, everything, as nearly as I could ascertain, was normal.

As to treatment, I don't think but little, if anything can be done in a case like this, by way of medication. Dr. Scott, in *American Journal of Obstetrics,* thinks that hysterical patients should not go without treatment. Dr. Gehrung, in same discussion thought they required but little, if any treatment. As for my part, taking a unique case like this, I am inclined to agree with the latter. I found that pointed, positive, and almost harsh language was required to control this patient, mild and sympathetic words from different persons in the room, only seemed to aggravate her condition. When the room was full of visitors she would behave much worse than when left alone—hence by excluding all callers, I had the satisfaction of seeing her out much sooner than I expected.

